# Longitudinal epigenetic and gene expression profiles analyzed by three-component analysis reveal down-regulation of genes involved in protein translation in human aging

**DOI:** 10.1093/nar/gkv473

**Published:** 2015-05-14

**Authors:** Marc Jung, Seung-Gi Jin, Xiaoying Zhang, Wenying Xiong, Grigoriy Gogoshin, Andrei S. Rodin, Gerd P. Pfeifer

**Affiliations:** 1 Department of Cancer Biology, Beckman Research Institute, City of Hope, Duarte, CA 91010, USA; 2 Center for Epigenetics, Van Andel Research Institute, Grand Rapids, MI 49503, USA; 3 Department of Diabetes and Metabolic Diseases Research, Beckman Research Institute, City of Hope, Duarte, CA 91010, USA

## Abstract

Data on biological mechanisms of aging are mostly obtained from cross-sectional study designs. An inherent disadvantage of this design is that inter-individual differences can mask small but biologically significant age-dependent changes. A serially sampled design (same individual at different time points) would overcome this problem but is often limited by the relatively small numbers of available paired samples and the statistics being used. To overcome these limitations, we have developed a new vector-based approach, termed three-component analysis, which incorporates temporal distance, signal intensity and variance into one single score for gene ranking and is combined with gene set enrichment analysis. We tested our method on a unique age-based sample set of human skin fibroblasts and combined genome-wide transcription, DNA methylation and histone methylation (H3K4me3 and H3K27me3) data. Importantly, our method can now for the first time demonstrate a clear age-dependent decrease in expression of genes coding for proteins involved in translation and ribosome function. Using analogies with data from lower organisms, we propose a model where age-dependent down-regulation of protein translation-related components contributes to extend human lifespan.

## INTRODUCTION

Aging can be defined as a multifactorial and time-dependent decrease of functions. The scope and interplay of various aging aspects, mostly derived from model organisms such as *Caenorhabditis elegans* (1), are still insufficiently understood. For studying mammalian aging, it became *de rigueur* in the recent literature to apply large-scale (so-called ‘omics’) approaches. These were mainly focused on transcriptomics and DNA methylation (2,3). One insight derived from these studies was the emergence of an age signature largely independent of tissue type with regards to transcriptional changes (4) as well as DNA methylation changes (5). However, as recent multiple tissue comparison studies suggested, gene expression and methylation changes can also be tissue-specific (6,7).

So far, mainly cross-sectional study designs with sample sizes ranging from 30 to >800 have been applied to quantify age-related changes (6,8–11). The obvious shortcoming of such approaches, compromising the biological meaning of the analysis, is the potentially significant inter-personal variation. These variations, in for instance DNA methylation patterns, are caused by genetic and environmental factors (12,13). Furthermore, the ‘standard,’ well-established data analysis tool for identifying and quantifying age-related changes has been, up to now, multivariate linear regression (14). While sufficiently robust and easy to implement and interpret, it has a limiting explicit assumption of linearity of age-related changes; but it is not yet clear if aging can be modeled exclusively by gradual changes. As another consequence, multivariate linear regression has difficulty combining potentially predictive data of varying distributional nature (heterogeneous data types). Longitudinal studies, where the same individual is followed over time, are preferred inasmuch as they are not confounded by inter-personal variation. However, sample sets available for longitudinal studies are rare and often the sample number is limited.

Most previous studies were focused on either transcriptional or DNA methylation changes with age (2,4,15–19). However, other epigenetic factors (such as histone modifications) are also important (20) but have rarely been investigated in a genome-wide context (21), although a tangible link between histone methylation and longevity in *C. elegans* and *Drosophila melanogaster* has been established (22–24). Building on that, we wanted to gain more insight into two processes: whether genome-wide age-related epigenetic changes follow a specific pattern (as opposed to occurring randomly); and whether alterations brought about by DNA methylation and histone modifications are linked to transcriptional changes as opposed to non-functional, random accumulated age-related epigenetic changes. DNA methylation changes in CpG islands (CGIs) in mouse intestine are an example of non-random changes. These changes could be validated as one effect of aging for a selected group of regions, supporting epigenetic deregulation (18).

In this study we detail what, to the best of our knowledge, is the first longitudinal and integrative transcriptional and epigenetic aging study. Incorporating transcriptional, H3K27me3, H3K4me3 and DNA methylation changes and making use of implicitly non-parametric gene set enrichment data analysis, we put special emphasis on our novel analysis framework. Using a limited set of 10 longitudinal aging sample pairs, we developed a novel analysis method, called three-component analysis (3CA), which considers the signal intensity of specific genes and the variance of the signal among all sample pairs in addition to the temporal changes measured to arrive at a single value for gene ranking of the most significant age-associated differences. Data analysis approaches of this nature are common in computer science and statistics, ranging from dimensionality reduction/feature selection (construction) to principal component analysis to unsupervised machine learning (clustering) (25,26). However, while they are fitting to the problem in question, to the best of our knowledge they have not been used in the biological research area so far. The method we propose below is closest to the ‘feature construction’ concept, as defined, for example, in (26). At this point we should notice that, while primarily data-driven and mathematically rigorous, our approach also relies on our understanding of the relative importance of biological aspects, thus avoiding the typical initial data exploration/feature selection step common to many data mining strategies. In a sense, we attempted to incorporate expert knowledge in an otherwise ‘objective’ analysis framework.

Our starting materials were short-term propagated fibroblasts from skin biopsy samples derived from the Baltimore Longitudinal Study of Aging (27). This tissue source provided a homogeneous cell population thus circumventing problems, which normally arise due to a bias in different cell compositions such as found in blood or directly processed skin biopsies (28). Using this novel analysis approach and unique study design, we found that the single most important component contributing to intra-individual variability in aging profiles is the functional cluster of protein translation-related processes.

## MATERIALS AND METHODS

### Cell culture

The human skin fibroblast cultures from 10 individuals of varying ages from 35 to 75 years old, established from skin biopsies taken from the inner part of the upper arm of young and old male donors, were obtained at low-passage number from the National Institute on Aging, the Aging Cell Repository (Coriell Institute for Medical Research, Camden, NJ, USA). See Supplementary Table S1 for a detailed list of samples. Cells were maintained in Dulbecco's modified Eagle's medium supplemented with 10% fetal bovine serum and without antibiotics at 37°C in a 5% CO_2_ standard incubator. To conduct an integrated analysis of transcriptional and epigenetic changes, and to avoid cell culture-associated changes, each cell sample was prepared once for all downstream assays.

### RNA-seq

Total RNA from human skin fibroblasts was prepared using PureLink RNA Mini kit (Invitrogen; Carlsbad, CA, USA). The cells were lysed by addition of lysis buffer, the lysate was then homogenized using QIAshredder (Qiagen; Valencia, CA, USA) and centrifugation at 12 000 xg for 2 min and then we followed the manufacturer's protocol. The quality of the prepared total RNA was evaluated using an Agilent 2100 Bioanalyzer (Agilent; Santa Clara, CA, USA). cDNA libraries for RNA-seq using oligo d(T) primer were prepared from RNA samples with an RNA integrity number of >8.5. The RNA-seq data were generated on an Illumina HiSeq 2000 instrument. Expression of specific genes was validated by RT-PCR.

### Mapping of 5mC

Enrichment of the methylated DNA fraction by the methylated-CpG island recovery assay (MIRA) was performed as described previously (29). In brief, the methylated fraction of sonicated genomic DNA from human skin fibroblasts was captured using recombinant MBD3L1 and MBD2b proteins. Ligation-mediated PCR was performed to amplify MIRA-enriched DNA. Nimblegen tiling arrays were used for analysis of 5mC genomic distribution (720k human CGI plus promoter arrays). The labeling of amplicons, microarray hybridization and scanning were performed according to the Nimblegen protocol. These arrays cover all UCSC Genome Browser-annotated CGIs and the promoter regions of all RefSeq genes. The promoter region covered is from −2440 to +610 bp relative to the transcription start sites (TSSs). For all samples, the MIRA-enriched DNA was compared to input DNA.

### Combined bisulfite restriction analysis and bisulfite sequencing

In total, 0.5 μg of each genomic DNA was treated with sodium bisulfite and the obtained PCR products were subjected to combined bisulfite restriction analysis (30) and sequencing analysis. Bisulfite-modified DNAs were PCR-amplified (primer sequences available on request). After PCR, the PCR products were cloned and sequenced directly, or they were digested with the BstUI restriction enzyme New England Biolabs (NEB), which cleaves only methylated DNA after bisulfite conversion. The digested PCR products were separated by electrophoresis on 2% agarose gels.

### Chromatin immunoprecipitation

Human fibroblasts were cross-linked in 1% formaldehyde solution, and then we followed methodology as described (31). Four micrograms of protein–DNA complex was used for each IP. Chromatin was immunoprecipitated with antibodies against histone H3K27me3 (Millipore, #07–449), or with anti-histone H3K4me3 antibodies (Active Motif, #39915). The procedure for DNA amplification after chromatin immunoprecipitation (ChIP) followed the protocol as described previously (29). Nimblegen promoter and CGI tiling arrays were used for the histone modification profile analysis. ChIP DNA fractions were compared against input DNA. Data were extracted from scanned images by using NimbleScan 2.3 extraction software (Nimblegen Systems).

### Methylation and RNA QC and pre-processing

For RNA-seq analysis, we obtained RPKM values for all genes and transcripts, which were calculated by using cuffdiff (32). We further corrected genes for potential CG bias by conditional quantile normalization (33). For annotation, we used the latest GENCODE build (34) as a reference for aligning RNA-seq related reads. Thus we obtained a total of 55 753 gene counts and 261 771 transcript counts. For single gene analysis, we used the gene-wise RPKM values matched to gene symbols, defined by GENCODE. For transcripts we calculated for each internal ID the matching transcript with the minimal 3CA score so that the transcript IDs would result in a list of unique gene IDs. The expression data from Shanley *et al*. (35) was obtained from the GEO repository (accession number GSE8121). The Affymetrix arrays were processed as described by Shanley *et al*. (RMA processed, then normalized to mean of controls) (35), and for 3CA analysis we used the respective pairs of septic shock day 1 and day 3. Those Affymetrix probe IDs with a 3CA score lower than one standard deviation (SD) from the lowest value were converted to gene symbols and used for functional enrichment analysis with DAVID (36,37). If not otherwise mentioned, subsequent analysis steps were done with R and Perl scripts.

For Nimblegen based promoter array analysis, samples were quantile-normalized and arrays were normalized by Nimblegen's recommended method. Log2 probe ratios were binned in 500 bp windows with 250 bp step size with at least four probes per window. To prevent the inclusion of log changes in the lower quantiles below 50% and including the cases of higher input signals than enrichment signals after normalization, values were reset to the 50% quantile values and windows with none or one probe above the 50% quantile were discarded. We converted the RefSeq IDs of the design template to the corresponding best matching Ensemble transcript ID.

### 3CA-guided GSEA

The details of the 3CA algorithm can be found in Supplemental Experimental Procedures. Including the sample signal strength for both the promoter arrays and the RNA-seq derived data ensured that low intensity transcripts and lower ratio changes, which we associated with lower confidence given the smaller sample size, were not present in the top ranked lists.

As a general means of choosing top ranked scores, we transformed the 3CA scores to the distance from the top scored target (the minimum of the 3CA score) to the current target in SD units and chose a cutoff of 1 SD for all sets. The top 2% quantiles were included within the 1 SD cutoff criteria.

Signed scores for temporal variance could be directly used as a ranked list file format. For gene set enrichment analysis (GSEA) we ran javaGSEA v2.0.13 on the acquired pre-ranked gene lists with the default parameters. As gene sets we either used our predefined gene sets obtained from the functional clusters of the DAVID analysis and the top scored genes or all curated functional annotations from MsigDB (c2.all.v4.0.symbols.gmt).

### Functional term enrichment analysis

We performed gene ontology analysis by using DAVID functional annotation tools with Biological Process FAT, Molecular Function FAT, Reactome and Kegg Pathways, SP_PIR_KEYWORDS as well as SMART, INTERPRO, EC_NUMBER and UP_SEQ_FEATURE terms.

For transcription factor binding site evaluation, we used UCSC_TFBS. For the Nimblegen promoter arrays, we used all genes from the tiling array as a background set. For the epigenetic sets, we used all RefSeq identifiers from the array design as a background. For GSEA we mapped all transcript variants obtained from cuffdiff to their related gene symbols. If more than one transcript mapped to a gene symbol, we chose the one with the higher temporal change (t component of the 3CA algorithm).

For functional analysis of top scored CGIs we used GREAT (38). CGIs were obtained from the USCS table browser, using the NCBI36 assembly, filtered for overlap with Nimblegen array promoter tiling regions and size-filtered for at least 500 bp. As background we chose the obtained total set of CGIs.

### Distance-related temporal analysis and functional cluster analysis

CGIs and CGI shore regions were binned into 500 bp windows with a step size of 250 bp. Binning for shore regions started at the CGI boundary and then continued with 250 bp distance increments. For relating binning windows to CGIs, we always chose the position closer to the next CGI. Recalculated, averaged and filtered probe ratios were taken as input for 3CA. Corresponding v, s and t values were calculated for all CGI regions and shore regions and partitioned according to distance to the closest CGI boundary.

### Data access

The GEO accession numbers for epigenetics data and for the expression data are GSE51517 and GSE51518, respectively.

## RESULTS

### Global transcriptional profiling and paired sample statistics

**Figure 1.**
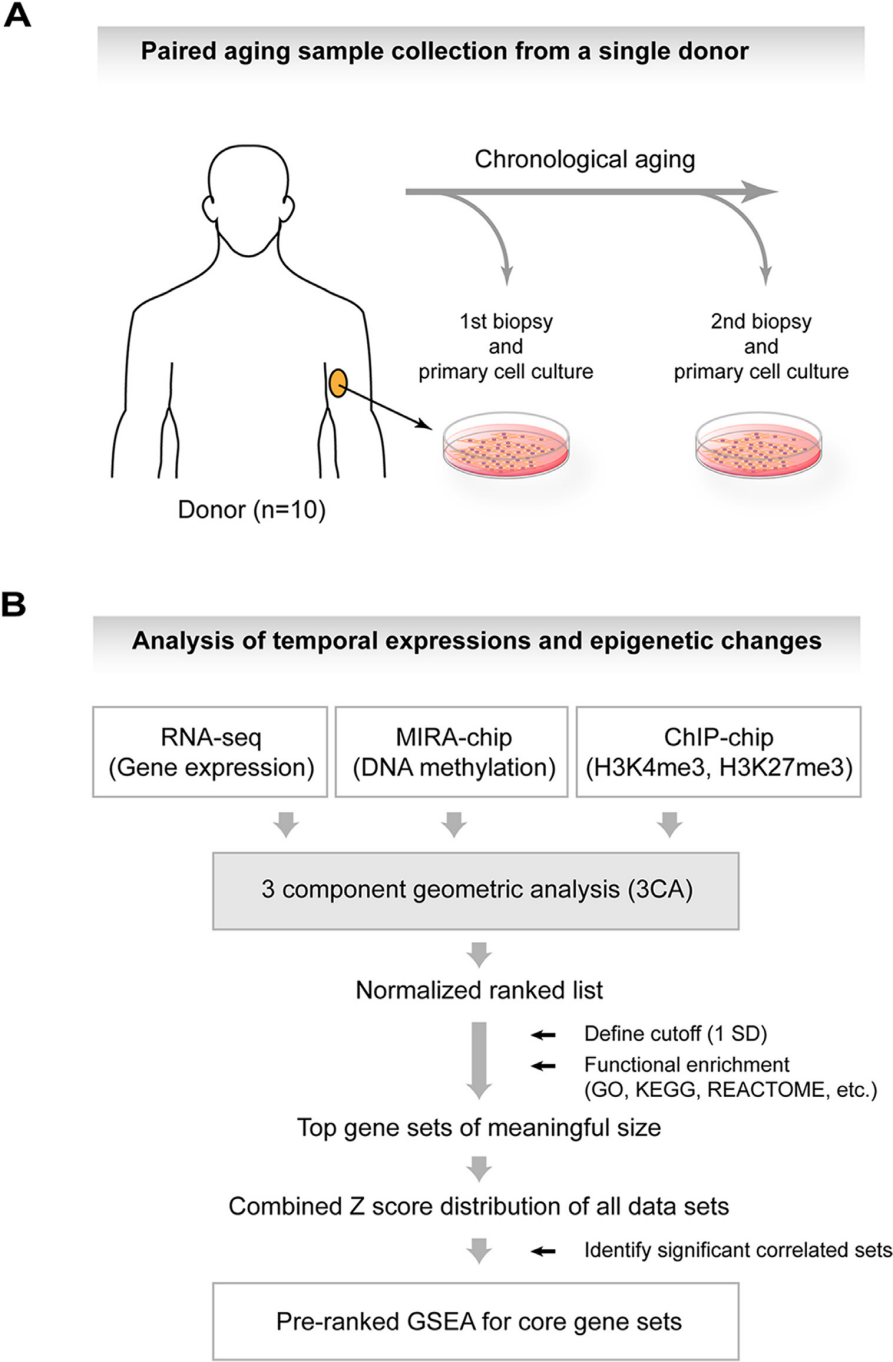
Schematics of the GSEA-guided 3CA analysis framework. (**A**) Illustration of the experimental design used for the study. (**B**) Overview of the experimental and data analysis steps.

Twenty low-passage dermal fibroblast primary cultures obtained from the inner side of the upper arm were chosen from 10 sample pairs and were subjected to Illumina HTseq 2000 RNA-sequencing. Samples were from two consecutive age time points separated by 12–19 years (Figure 1A and Supplementary Table S1). Gene- and transcript-specific expression values were calculated (32) and gene values were corrected for potential GC-content bias (33).

In order to derive time-dependent signatures, we first applied a paired statistic by calculating the intra-individual changes. We compared intra-individual with inter-individual expression changes, where we considered an age difference of at most 2 years for the latter, which resulted in a background set of 10 inter-individual pairs. In this way, we obtained two groups of intra- and inter-individual expression changes and could determine statistically significant differences. After standard false discovery rate (FDR) multiple testing correction (39), only a few genes could be selected as significantly correlated with intra-individual time changes (Supplementary Figure S1A). As GSEA offers a higher sensitivity approach for detecting signature changes for predefined gene sets (40), we applied our modified GSEA algorithm to the data, resulting in a higher number of significant gene sets (Supplementary Figure S1B). Functional gene sets obtained via standard analysis techniques (e.g. unsupervised learning/clustering) generally failed to detect important processes such DNA repair, extracellular matrix and cell-cycle-related terms, which are age-related (Supplementary Figure S1C). This gave us the motivation to develop a new method aimed at providing a more realistic model fit.

### Estimating age-related changes by applying a constrained three-component selection filter

**Figure 2.**
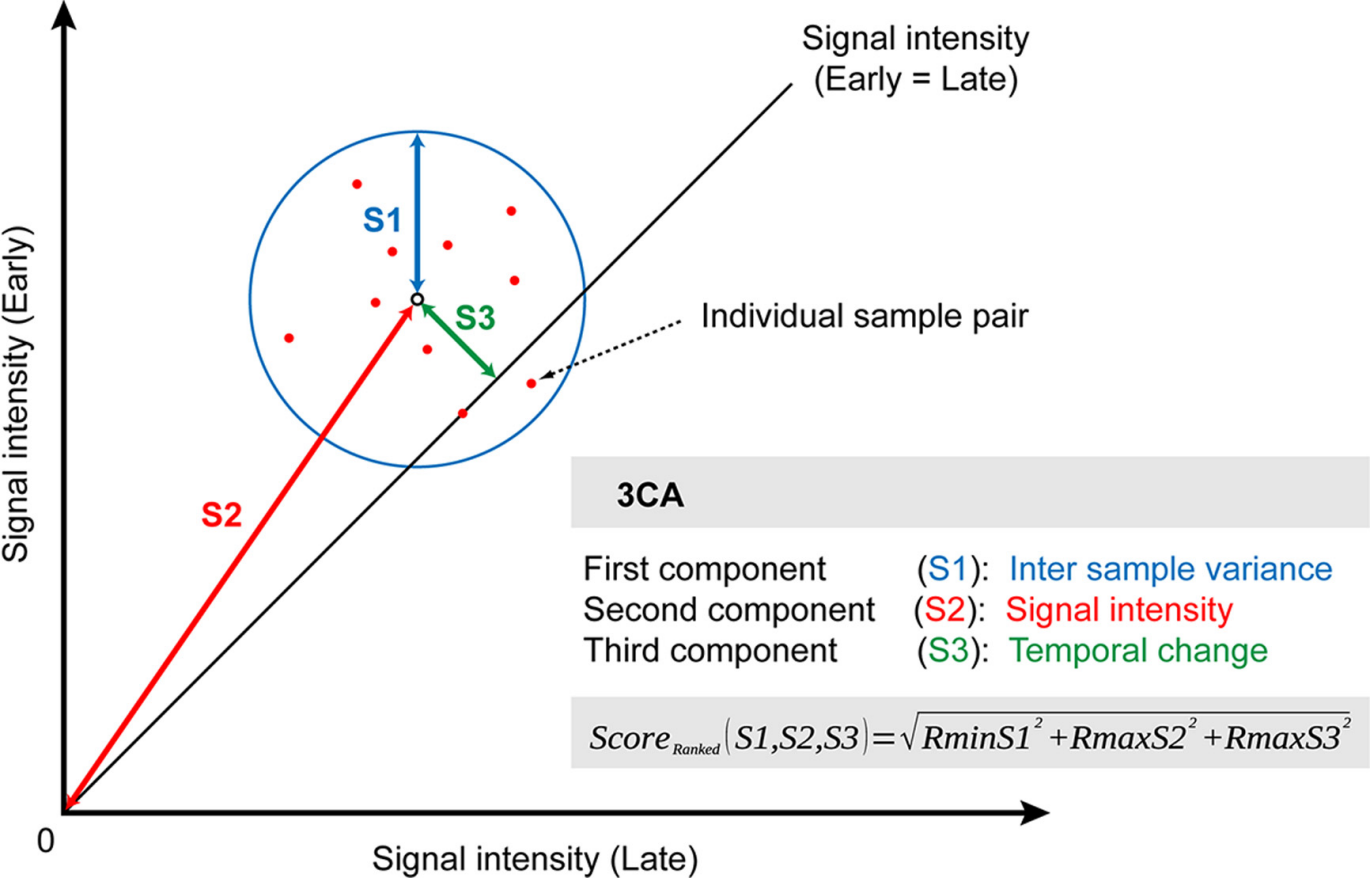
Graphical representation of the 3CA approach. Individual sample pairs can be visualized as dots in a plane. Significant age-related changes can be identified as an optimal intersection of signal intensity, sample variance and temporal change.

Our main goal was to determine if we could derive significant age-related functional terms by maximizing the signal obtained from the interplay of the paired design and the corresponding enrichment in biological functional gene sets. In essence, we pursued a data analysis approach which would guard against overparametrization/overfitting (i.e. overestimating the significance of random patterns, a common pitfall when analyzing small sample size/large number of potentially predictive variables in data sets) and yet assure a good model fit for the observed pairs, thus implying predictive modeling capabilities. This was the rationale behind developing and implementing an algorithm that we will refer to as the 3CA (Figure 2). The goal was to derive a single integrative metric for temporal, age-related changes for all analyzed samples (the ‘3CA score’). After evaluating various modeling alternatives, we came to the conclusion that a vectorized form in a two-dimensional plane would be, while not incurring any information loss, the most compact model representation (a technique generally known as feature construction) (26).

The three components comprising the model are: (i) the inter-individual variance, derived by calculating the minimum distance for all normalized contrasts/pairs (e.g. young and old), resulting in a centroid with a radius approximating the variance, (ii) the absolute signal strength, which would correct for too low signals and could be visualized as the length of the vector from the coordinate origin to the centroid and (iii) the temporal distance, defined as the shortest (perpendicular) distance between the centroid and the 45 degree reference line (reflecting the null hypothesis, i.e. signal intensity remaining constant). The two attractive aspects of this approach are (i) no explicit (distributional) or implicit assumptions regarding outliers, and (ii) the ability to combine the above three measures into a single score/metric. We should emphasize here that the three components were chosen based on biological considerations. Starting with the empirical distribution of variance, derived from the ‘integral’ representation of the intra-individual and subsequently ranked temporal changes, we were able to calculate a significance threshold (cutoff value).

Importantly, we want to stress that 3CA by itself is not a statistical (stochastic) method but rather a deterministic one, a species of feature selection (construction) approach, specifically a constrained component selection algorithm. Robustness is achieved by constrained optimization of the three components and not by permutation. For example, if time-dependent change and signal intensity of two genes have the same contribution, the gene with the lower variance will be assigned a higher-ranking score. By using an equal weight function we are not making any assumptions about the data. Any change of the weight function would require more detailed hypothesis- or knowledge-driven assumptions, whereas our only assumption at this time is the importance of the three components. To test the general validity of our method, we analyzed a published longitudinal microarray expression data set, consisting of 30 sample pairs, derived from a 48 h sepsis time course in children (see the Method section) (35). We identified indeed functional terms strongly related to systemic inflammatory reactions without using any statistical methods (Supplementary Table S2).

Using the 3CA approach, we established a ranked list of the top ∼500 genes with age-dependent changes (Supplementary Table S3). To show that all three components (notably temporal changes) are important for identification of significantly altered genes, we calculated the percentage of contribution for each component via computing the inverse ranks for all component scores and then calculating the percentage for each gene (see Supplementary Experimental Procedures). Thus, a hypothetical gene with equal contribution from all the three components would show a roughly 1/3, 1/3, 1/3 component contribution ratio.

We compared the actual distribution for each component for the top 200 3CA-scored genes, for randomly chosen 200 genes (random) and for the top 200 scored genes with randomly permuted samples (shuffled/control). Supplementary Figure S2 summarizes the results of the analysis. The contribution of temporal changes as a factor essentially remains the same. This is the expected behavior in the context of our design (equally weighted components), and there is no substantial bias for the top 200 genes. The primary reason we chose to weight the components equally was that we had no biological justification to do otherwise, and so we settled on the most ‘agnostic’ model. Component weighting can be easily incorporated in the modeling framework when biologically motivated.

### Single skin aging-specific genes

Considering that we used fibroblasts as a cell type, we first asked if there were age-related changes in gene expression that would be tissue-specific. This led us to the supposition that a closer look at extracellular matrix-related genes would be informative in the context of the microenvironment of the dermis. Indeed, we detected a set of metallo-proteinases (*MMP2*, *MMP14*), collagens (e.g. *COL6A1*, *COL3A1*, *COL5A1* and others) and elastin (*ELN*), which showed a significant longitudinal change in expression in addition to being closely correlated to skin aging (Supplementary Table S3) (41,42). We observed statistically significant down-regulation, in all sample pairs, for the elastin (*ELN*) gene (Supplementary Table S3; Supplementary Figure S3). The highest scored transcript we detected with our algorithm mapped to the up-regulation of *CD63*, a lysosomal membrane protein gene. We also detected the tendency for up-regulation of lamin A (*LMNA*), which is a gene contributing to progeria and aging (43) (Supplementary Table S3). Next, we were interested in gaining information for whole gene sets in order to capture the most relevant functional terms changing with age.

### Combining 3CA with pre-ranked GSEA

The resulting 3CA score suggests the directionality of change for a transcript, but it would not necessarily indicate whether a gene set as a whole is up- or down-regulated. As we have ranked temporal changes for all transcripts, GSEA provided complementary statistical support for the directionality of the changes. After applying 3CA, we generated a ranked list of scores with lower values indicating higher significance. For combining the 3CA score with GSEA, we obtained the functional clusters from the selected entries at the top of the list as derived by using functional annotation software, e.g. DAVID (37). We concentrated on the transcripts that had the top 2% 3CA scores within the margins of 1 SD around the minimum so that we had a high-confidence set of transcripts even before the subsequent functional enrichment analysis (Figure 1B). For the corresponding gene IDs, we would thus use those temporal changes which were bounded by an upper limit of the variance and which could take positive or negative values, for ranked GSEA.

### Age-related functional clusters are largely defined by factors involved in translation

Starting with functional enrichment of the top scored genes with potential age-related functions, the strongest signal we observed was for the keyword ‘acetylation’ (Supplementary Table S4). Though ‘acetylation’ is a somewhat generic term, it has been strongly linked with aging before (20). When ascertaining the top-ranked biological process-related functional clusters of age-related transcriptional changes, it quickly became strikingly obvious that the most enriched clusters were all connected to protein translation including ribosomal proteins, translational initiation and elongation, and protein transport-related factors (Supplementary Table S4). Biological processes such as protein degradation (e.g. proteasome and ubiquitin-related ones) showed high enrichment as well, and so did factors involved in ATP synthesis. As we wanted to further rule out that the discovered functional sets were artifacts due to erroneously prioritized signal intensity, we repeated the analysis without including the signal intensities. Even in the absence of signal intensity, we could recapture the functional terms we discovered with all three components (Supplementary Table S5). We still included the intensities in the final score, as it would guard against overemphasizing single gene examples with low variance and low signal intensities.

**Figure 3.**
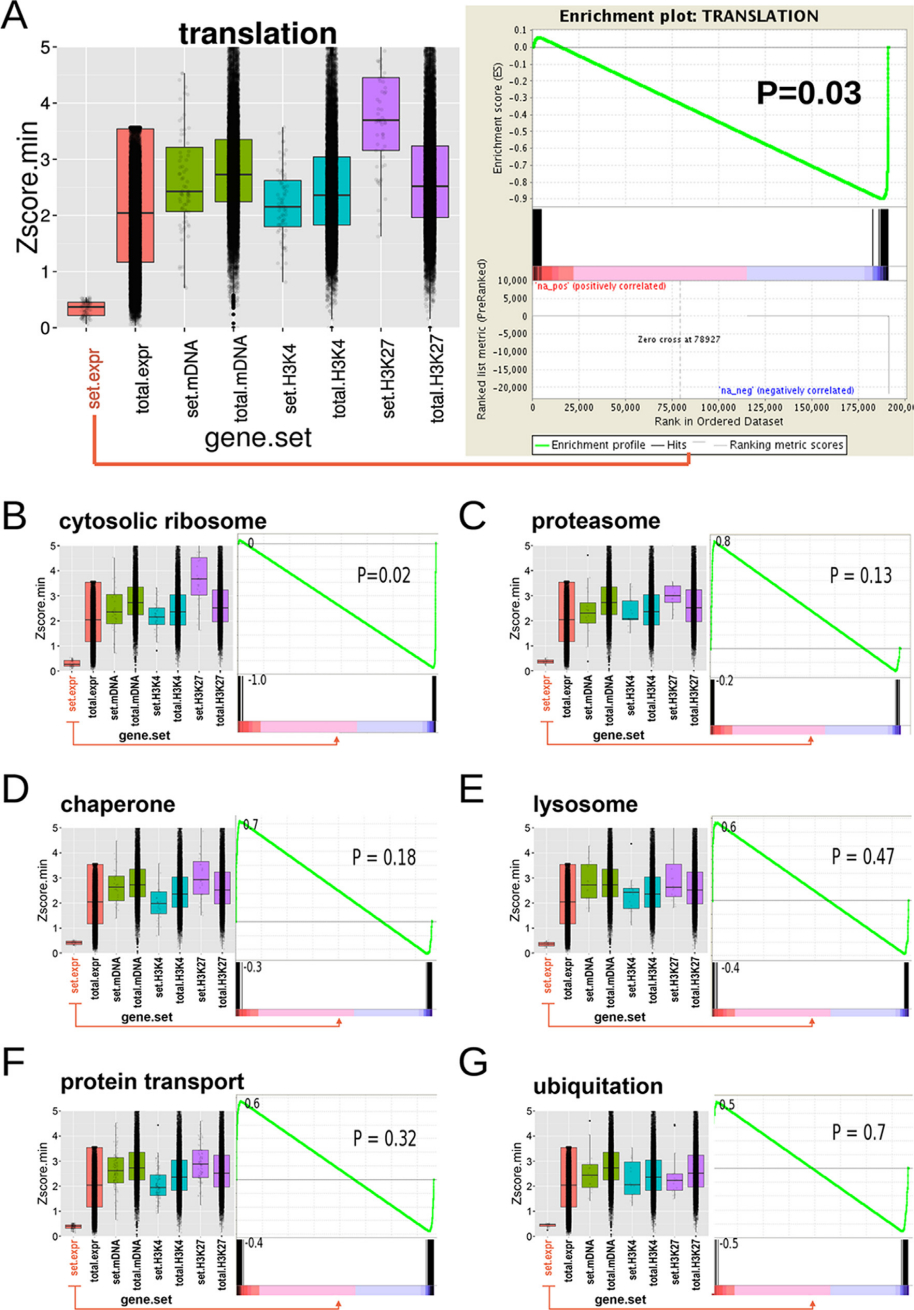
3CA-guided GSEA detecting the most significant gene sets correlated with temporal up- or down-regulation. Temporal expression and epigenetic changes can be evaluated in one plot. For a comprehensive view, we plotted the 3CA score distributions for gene expression (RNA-seq), DNA methylation (meDNA), H3K4-trimethylation (H3K4me3) and H3K27-trimethylation (H3K27me3) for the specific gene sets (set) and for all genes (total) (left panels). In addition to the box charts, we plotted a scattered distribution of the scores (right panels). In this example, we examined the significant 3CA scores for gene expression for matched promoter regions of the epigenetic sets. In order to access the significance for up- or down-regulation, we obtained the temporal changes for the specific gene sets and performed GSEA with the obtained pre-ranked list (40). As we pre-filtered for significant 3CA scores, the respective distribution of temporal changes will be pushed to the edges of the GSEA plots. The values at the y-axis of the enrichment plots are the maximum and minimum enrichment score values obtained from the temporal changes. Shown are all identified processes connected with protein turnover. **A**: translation, **B**: cytosolic ribosome, **C**: proteasome, **D**: chaperone, **E**: lysosome, **F**: protein transport, **G**: ubiquitination.

In order to determine if a whole functional group was related to up- or down-regulation, we ran pre-ranked GSEA analysis, using the temporal distances we had calculated before. According to the levels of enrichment obtained by DAVID analysis, for this purpose we grouped the main functional terms into the most relevant categories. Interestingly, out of all enriched gene clusters we identified for the top candidate sets, a significant correlation with down-regulation could only be shown for the ones related to translation (Table 1; Figure 3). It should be noted that our methodology also detected other protein homeostasis related functional terms (processes involving protein transport, chaperones, lysosomes and ubiquitin) as significantly temporally changed. In most cases the directionality for the gene set was not as strongly tending toward down-regulation (Figure 3). In general, we did not detect any significant gene sets associated with up- or down-regulation without a preceding ‘filtering’ for 3CA scores. Thus, we conclude that for this specific aging-related expression data set, a pre-filtering for significant 3CA scores, reflecting temporal changes, is essential.

**Table 1.** Overview of top identified categories

		GSEA	GSEA	3CA	3CA	3CA	3CA
NAME	SIZE	NES	*P*-val	*P*-val (expr)	*P*-val (mDNA)	*P*-val (H3K4me3)	*P*-val (H3K27me3)
							
**Tendency up-regulated**							
MRNA_SPLICING	80	1.61	0.03	4.98E-020	0.38	<0.01	0.80
PROTEASOME	37	1.33	0.13	3.42E-011	0.02	0.32	0.98
CHAPERONE	56	1.25	0.18	2.81E-015	0.17	<0.01	0.99
PROTEOGLYCAN	35	1.15	0.29	1.59E-006	0.54	0.30	0.65
RESPONSE_TO_NUTRIENT	42	1.13	0.31	5.06E-013	0.23	0.02	1.00
PROTEIN_TRANSPORT	236	1.07	0.32	6.68E-061	0.03	<0.01	1.00
EXTRACELLULAR_MATRIX	144	1.06	0.36	1.92E-030	0.82	0.22	0.93
LYSOSOME	49	0.99	0.47	6.94E-012	0.47	0.23	0.89
RESPONSE_TO_OXIDATIVE_STRESS	65	0.96	0.52	1.64E-018	0.28	0.01	0.95
UBIQUITIN_MEDIATED_PROTEOLYSIS	46	0.82	0.70	2.91E-013	0.05	0.16	0.10
**Tendency down-regulated**							
CYTOSOLIC_RIBOSOME	81	−1.63	0.02	1.64E-028	<0.01	0.03	1.00
TRANSLATION	180	−1.62	0.03	3.74E-054	0.02	0.01	1.00
APOPTOSIS	163	−1.37	0.12	6.47E-038	0.27	0.01	0.93
ANTI_APOPTOSIS	66	−1.32	0.15	4.26E-017	0.55	0.21	0.99
ELECTRON_TRANSPORT_CHAIN	46	−1.29	0.17	1.57E-014	0.19	0.22	0.98
MITOCHONDRION	186	−1.23	0.19	1.44E-056	0.28	<0.01	1.00
AGING	34	−1.11	0.34	3.19E-009	0.24	0.56	0.99
FOCAL_ADHESION	143	−0.94	0.50	4.79E-027	0.41	0.04	0.98
CELL_GROWTH	34	−0.94	0.55	3.54E-009	0.11	0.15	0.68
CYTOSKELETON	177	−0.91	0.56	1.37E-032	0.06	<0.01	0.92

Top score functional sets (within 1 SD) with GSEA enrichment scores and Wilcoxon *P*-values for expression and epigenetic changes.

One underlying biological mechanism is that decrease in translation is a consequence of reduced mitochondrial activity and energy production; however, while there were components of the electron transport chain linked to down-regulation, there was no clear tendency for the whole set.

### Age-related functional clusters that include epigenetic marks

It has been proposed that ‘epigenetic drift,’ i.e. the deterioration of epigenetic marks on specific genes is a component of the aging process (17,19,44,45). In order to combine RNA-seq data with DNA methylation data in the same samples, we employed the methylated-CGI recovery assay (MIRA), which has been proven to be a reliable and sensitive method for obtaining genome-scale methylation data (46). For histone modifications, we added H3K4 trimethylation ChIP-chip for active or poised promoter regions and H3K27-trimethylation ChIP-chip for Polycomb-repressed regions, and then focused on the promoter areas.

**Figure 4.**
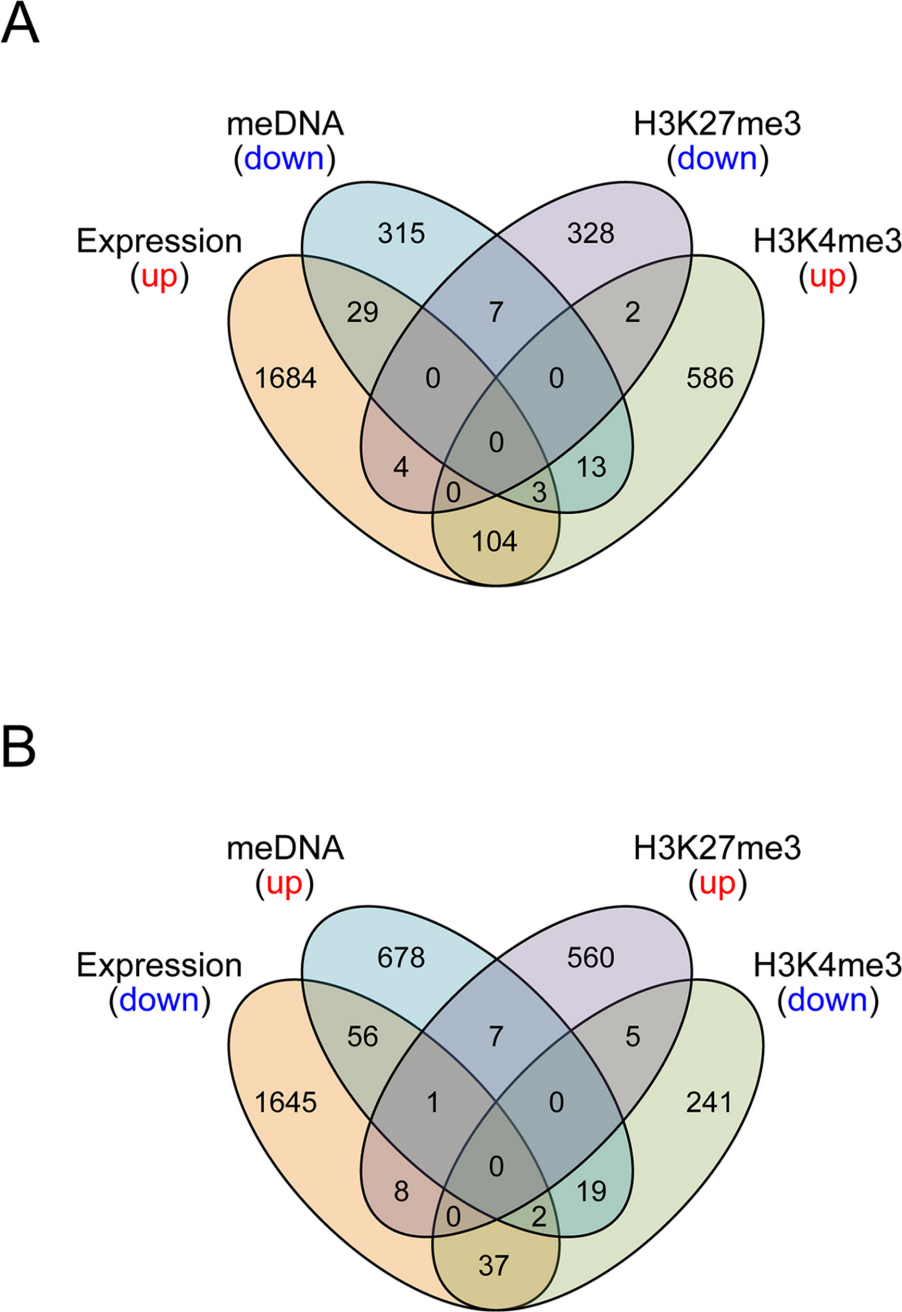
Venn diagrams showing overlap between epigenetic and transcriptional data sets for the top 3CA scores. Transcripts which were preferentially up-regulated with age were compared with down-regulated H3K27me3 regions, down-regulated DNA methylation regions, and up-regulated H3K4me3 regions and vice versa (panels **A** and **B**, respectively). Data included are within 1 SD from the best score (top ∼2%).

First, we looked for correlations between expression and the single epigenetic marks. For that purpose, we compared expression changes with epigenetic changes for the top scored genes within the 1 SD threshold. By discriminating between positive and negative temporal changes of the epigenetic marks relating to up- or down-regulation during aging, we found that temporal changes for H3K4me3 correlated with gene expression changes with 13% of down-regulated H3K4me3 marks correlating with reduced gene expression and 15% of up-regulated H3K4me3 marks correlating with increased expression. This correlation trend was followed by temporal changes of DNA methylation with 9% of all hypomethylated sites linked to increased gene expression and 8% of all hypermethylated sites linked to decreased gene expression (Figure 4). Surprisingly, changes in H3K27me3 did not significantly affect temporal changes of gene expression. In summary, H3K4me3 was the best predictor for gene expression, compared specifically to H3K27me3 and DNA methylation. One likely reason could be that regions for H3K27me3 were already occupied in the ‘early’ samples, which were derived from middle-aged males, and thus the signal intensity changes were too subtle to affect transcription. On the other hand, DNA methylation, H3K27me3 and H3K4me3 might not be sufficient to reliably predict time-related gene expression and a combination with other not tested factors may result in a stronger correlation.

In order to obtain the epigenetic scores, we split promoter arrays into 500 bp windows, allowing us to calculate average ratios for four tiling array probes and calculated average log-fold changes. The resulting values were used for calculating the 3CA scores, which then could be ranked to determine ‘top’ targets. As far as functional terms enriched for DNA methylation-associated temporal changes were concerned, though strong correlations were observed for developmental terms and an increase of DNA methylation at promoters (as well as proteolysis factors and down-regulation of DNA methylation with age; Supplementary Table S6), we did not detect significant clusters. As DNA methylation changes in *HOX* genes have been suggested to be involved in aging and cancer (5,47), we specifically looked for methylation-related changes at *HOX* clusters and, indeed, observed a distinct tendency for age-related changes of DNA methylation in genomic regions within the *HOXB* and *HOXD* clusters (Supplementary Figures S4 and S5).

Between transcriptional and epigenetic signal changes for functionally connected genes, the analysis toolkit and methodology proposed in this study can effectively serve as a statistically rigorous filtering/variable selection approach (48) aimed at detecting meaningful interactions. As such, we extended our test to the epigenetic data sets, first identifying all functional enriched terms and then using the related genes as *de novo* gene sets related to a specific set (e.g. genes related to the term UBL_CONJUGATION are enriched in DNA methylation). For the same gene set, for epigenetic and expression changes, we tested the 3CA score distribution. Interestingly, one gene set among all combinations, which showed an increase of H3K4me3 in promoters as well as an increase in gene expression, was related to DNA repair (Supplementary Figure S6C). Concerning the previously identified down-regulation of genes involved in translation, in terms of up-regulation of DNA methylation and down-regulation of gene expression, we did not observe a strong correlation; but we detected a gene set within the 1 SD threshold, which was related to translation (Supplementary Figure S6A).

### Region-specific epigenetic patterns

We also wanted to ascertain whether age-related changes were associated with specific genome locations. When looking at the TSSs of all genes used on the array, we noticed a tendency toward up-regulation of DNA methylation in close vicinity to the TSS, but no change in other epigenetic marks (Supplementary Figure S7, and data not shown). In order to test if age-related DNA and histone methylation changes were linked to certain positions in relation to promoter-proximal CGIs, we examined age-related methylation changes relative to CGI border positions (indicative of CGI shore regions) and observed a distinct trend for age-associated DNA hypermethylation at CGI regions. This effect became less significant when moving further away from the CGI borders and was not observed for the histone modifications. For H3K4me3, the age-related variance peaked at ∼500 bp from CGI borders and for H3K27me3 we did not notice any change in age-related variance for CGIs compared to the regions nearby (Supplementary Figure S7).

**Figure 5.**
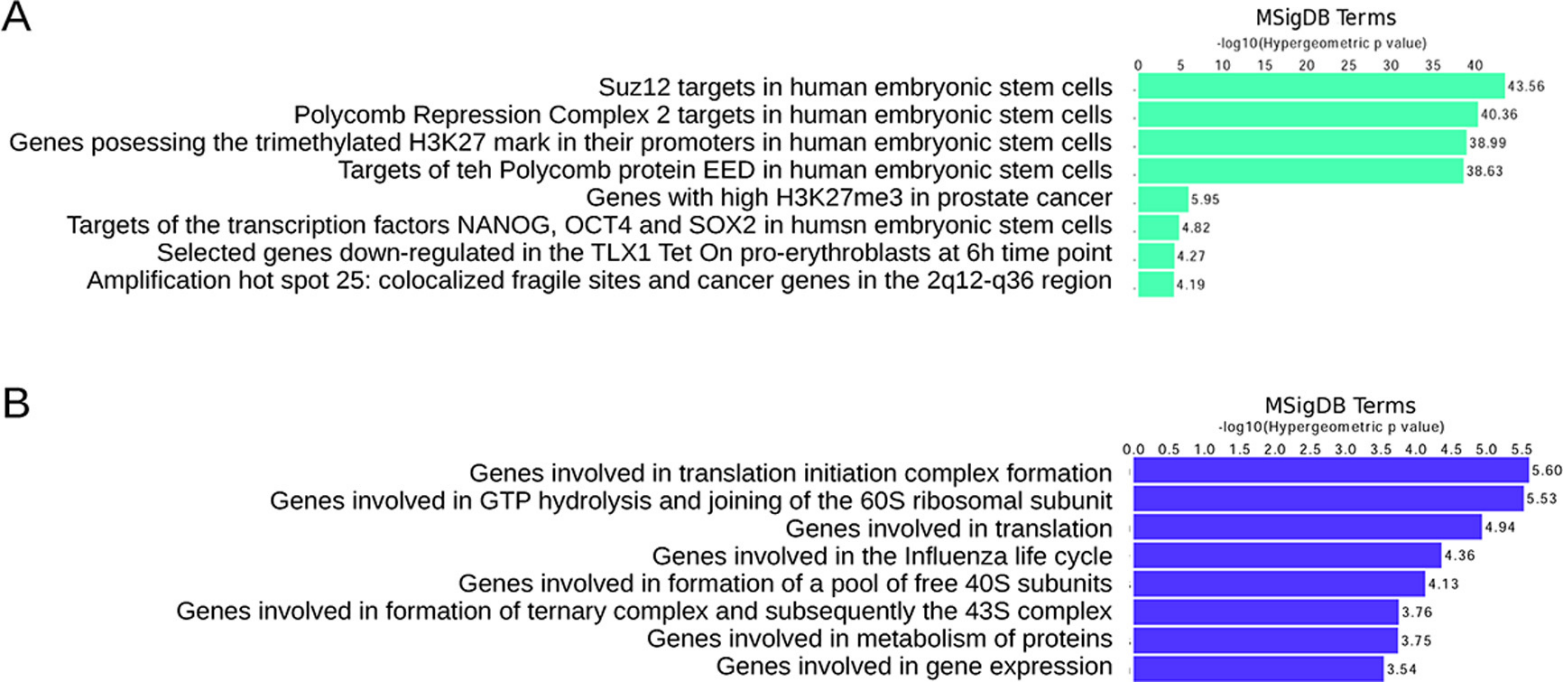
Functional enrichment analysis for CGIs. Data are for the top scored CGIs within 1 SD from the best score for both up- and down-regulated regions. Functional significance of top scored CGIs for age-related changes in H3K27me3 (**A**) and DNA methylation (**B**), defined by GREAT analysis and using terms of the Molecular Signature Database (MsigDB).

We asked if the most significant 3CA scores for CGIs would also coincide with functional correlations. Not surprisingly, the most significant temporal changes of H3K27me3 at CGIs were correlated with Polycomb targets in human embryonic stem cells (Figure 5A). Previously, it was observed that age-related DNA methylation is highly correlated with Polycomb target genes (3,5,18,49,50). Indeed, we found that a subset of homeobox genes showed a temporal change of DNA methylation (Supplementary Figures S4 and S5). In addition, hypermethylated CGIs were correlated with translation-related functional terms (Figure 5B). The genes *EIF3J*, *RPL27A*, *RPLP1*, *EIF3B* and *RPS20* were examples where 3CA scores were within the 1 SD threshold and down-regulated expression correlated with an increase in DNA methylation at CGIs.

In summary, between the three epigenetic marks analyzed, overall we did not observe very strong correlations. The best evidence in support of a specific pattern relative to CGIs was related to DNA methylation; and the best predictor for gene expression change was an age-related H3K4me3 change.

### 3CA-guided GSEA identifies most functional clusters involved in a tissue-independent signature

Previous studies of transcriptional changes in aging argued for the existence of a tissue-independent signature (4,51,52). Hence, we wondered if the functional clusters in our ‘top’ regions, identified by 3CA score ranking, were congruent with the above signatures. Indeed, at a functional level, we could capture most of the age-associated terms, such as extracellular matrix (Wilcoxon *P*-value for z-score difference: *P* = 1.92E-030), apoptosis (*P* = 6.47E-038), response to oxidative stress (*P* = 1.64E-018), lysosomes (*P* = 6.94E-012) and electron transport chain (*P* = 1.57E-014) (Table 1).

### A signature of FOXO transcription factors

Given a set of functional terms that appear to change with age, a question naturally arises: Is there a set of transcription factors, which might be correlated with these terms? To address this question, we divided the top-ranked age-related genes as derived by our 3CA algorithm into up- and down-regulated groups and estimated the quantitative enrichment of transcription factor binding sites in related promoters using one-sided Fisher's exact test (Table 2). When looking for age-associated transcription factor binding sites with DAVID, we identified as statistically significant transcriptional down-regulation candidates FOXO1 and FOXO4 (*P* = 9.6E-10 and *P* = 7.6E-11, respectively, after FDR multiple testing correction). Both *FOXO1* and *FOXO4* transcripts were expressed in fibroblasts but expression levels did not change significantly with age. Functional enrichment analysis for genes with promoter regions containing those combined putative FOXO1 and FOXO4 binding sites included mostly protein transport, translation and adhesion (Supplementary Table S7). Given that protein translation factors were among the most significant down-regulated categories and FOXO factors have been linked with aging and the mTOR complex involved in translational control (53–55), we asked if we could identify potential target genes in our expression data. Indeed, we detected significant down-regulation of translation initiation factors (*EIF4G2*, *EIFG1*, *EIF3E*, *EIF3L*; see Supplementary Table S2); however, we did not observe any mTOR-related transcriptional changes.

**Table 2.** Potential transcription factors regulating age-related target genes

Term	Count	*P*-value (Benjamini)

**TF binding sites for up-regulated genes**		
ELK1	614	6.25E-017
MAZR	303	3.43E-015
PAX5	668	5.21E-014
NRF2	388	2.69E-013
SP1	334	3.01E-013
ARNT	571	3.09E-013
AHRARNT	621	7.03E-013
YY1	785	3.97E-011
ROAZ	539	9.38E-011
**TF binding sites for down-regulated genes**		
MEF2	845	7.97E-017
SOX5	569	2.78E-012
HLF	480	4.42E-010
EVI1	885	5.28E-010
FOXJ2	727	1.63E-009
FOXO4	613	2.22E-009
CDP	668	6.69E-009
POU3F2	655	1.93E-008
FOXO1	478	1.87E-008

The list of the most enriched transcription factors in relation to top scored up- and down-regulated age-related transcripts was derived by motif analysis of promoter sequences, using the default settings of DAVID.

## DISCUSSION

To the best of our knowledge, the presented research is the first longitudinal study of aging involving high-throughput transcription and epigenomic data that utilized a paired design. As opposed to cross-sectional designs, within the serially sampled design framework we were able to ascertain the best candidates in terms of variance, signal intensity and age-specific changes simultaneously, thus utilizing the available information more efficiently. Starting with an RNA-seq approach, we assessed temporal expression changes of >120 000 transcripts and combined expression with epigenetic changes related to DNA methylation and H3K4- and H3K27-trimethylation. We have developed an integrated data-driven analysis procedure, which allows us to rank temporal expression and epigenetic changes and relate them in a functional context. For low sample numbers, no additional statistical tests are required and for larger sample numbers our filtering method offers a robust way for feature selection and subsequent statistical approaches, e.g. probabilistic networks or machine learning approaches.

Notably, the results of a recent review to identify the hallmarks of aging (56) showed overlap with our enriched sets largely in the area of epigenetic alterations but also suggested links to the loss of proteostasis and transcriptional changes in mitochondrial genes. A decrease of protein synthesis during aging could not be identified in human muscle cells (52). However, our data are in line with previous studies, predominantly involving *Saccharomyces cerevisiae*, *C. elegans* and *Drosophila melanogaster*, which linked aging to decreased protein synthesis (57,58). As inhibition of translation has been linked to life span extension in *C. elegans* through depletion of the translational regulator S6K and the Eukaryotic initiation factors (eIFs) eIF2β/iftb-1, eIF4E/ife-2 and eIF4G/ifg-1 (59), we wondered if similar regulatory pathways exist in humans. While we could not find any significant down-regulation of mTOR- or S6K-related transcripts, we detected the down-regulation of several translation initiation factors and ribosomal protein genes. Although speculative at present, we propose that down-regulation of protein translation-related factors might be a compensatory response to extend lifespan in humans/mammals rather than being just a consequence of aging. This reasoning is based on findings in *C. elegans* where knockdown of key factors involved in protein synthesis leads to extended life span, arguing for a well-conserved mechanism (59). Very recently, Hoffmann *et al*. showed that Myc haploinsufficient mice (Myc^+/−^) had increased lifespan and were resistant to several age-related pathologies (60). These authors found that the expression of ribosomal protein genes was coordinately reduced in Myc^+/−^ tissues. The largest category of genes directly up-regulated by Myc is involved in protein translation reflecting an ancient function of Myc (61). These data are very much in line with our findings on reduced expression of this category of genes in human aging as a potential mechanism of lifespan extension.

As to why these changes have not been detected in previous cross-sectional comparisons, we argue that such expression changes were under-represented in cross-sectional studies as they may be more prone to inter-personal variance whereas in our approach we were prioritizing for optimal combination of variance and signal intensity change. In conclusion, perhaps the most important result in the present study is our discovery that the majority of translation-related processes are down-regulated with age. A recent study by Schrack *et al*. further strengthened the claim that resting metabolic rates decline as a function of time (62). It remains to be seen if decreased transcription of translation-related protein genes is a consequence of decreased systemic metabolic rates with age and if age-related diseases such as insulin resistance, hypertension, cardiovascular disease, osteoporosis and atherosclerosis, which are all closely related to metabolic dysfunction (63), are reflected by a specific response to translational control as well. Decreased protein synthesis rates might also be a consequence of age-related signaling pathway changes, i.e. reduced levels of IGF1 during aging could affect synthesis via lack of mTOR stimulation (64–66). Not all cell types may be associated with decreased translation during aging. In fact, a recent study showed the inverse effect in hematopoietic stem cells (HSCs) (67), where maintenance-oriented functions such as DNA repair were shown to be down-regulated (as opposed to our soma cell results). HSCs are known to have a lower and tightly regulated protein synthesis rate (68). We suggest a model where the age-related balance of maintenance/anabolic processes is essentially bidirectional, leading eventually to both depletion of stem cells in old age (69) and a slow metabolic reduction for somatic cells.

When exploring potential transcription factors, which are correlated with translation-related terms, we discovered FOXO1 and FOXO4 as the most likely age-related candidates. FOXO transcription factors are presumed to be involved in the maintenance of cellular homeostasis during aging, and functional gene groups they have shown to be involved with (70,71) indeed reveal a substantial overlap with the age-related clusters we identified. FOXO transcription factors are relevant effectors of the insulin and IGF-1 signaling pathway associated with longevity in worms and flies (1,72) and represent potential candidate regulators for the identified age-related signature changes. Further studies are required to refine our understanding of the link between the transcriptional impact of FOXO factors on protein synthesis related genes and aging in humans.

In summary, a combination of novel secondary data analysis methods, coupled with our unique longitudinal study design, allowed us to generate a robust set of functional terms related to age-related changes in gene expression as well as for epigenetic alterations that could serve as a guideline for detecting biomarkers of aging. Using general model assumptions, we found the decrease of transcriptional levels related to protein translation to be the strongest functional indicator for age-related pattern changes.

Importantly, by using an extended set of complementary analysis techniques (ranging from purely ‘agnostic’ data-driven to literature-driven ontological ones), we were able to obtain robust signals from a relatively small set of individuals. Of particular note, our methods should be applicable to many other studies based on paired designs. While our approach is particularly useful for paired design studies with relatively low sample numbers, we emphasize that it could be used in studies with larger sample numbers as well. As an example, tumor progression studies, where for instance a primary tumor is compared to a metastatic lesion or to a recurring tumor, could benefit from our methodology to identify the most relevant functional gene sets within a particular cancer. Other possible experimental designs may include analysis of patients or experimental animals before and after treatment. There are many other scenarios where our approach may be applied. Arguably, the most attractive feature of 3CA analysis is that it is not limited to a particular data type and could integrate data as different as mutations, gene expression, levels of proteins or metabolites, or epigenetic marks, with the promise of including other, diverse data types in the future.

## SUPPLEMENTARY DATA

Supplementary Data are available at NAR Online.

SUPPLEMENTARY DATA
