# Supplementary material for: Longitudinal epigenetic and gene expression profiles analyzed by three-component analysis reveal down-regulation of genes involved in protein translation in human aging
Source: Nucleic Acids Res. 2015 May 14;43(15):e100. doi: 10.1093/nar/gkv473 (PMC4551908; doi:10.1093/nar/gkv473)
Supplement: SUPPLEMENTARY DATA [file supp_43_15_e100__index.html]

Longitudinal epigenetic and gene expression profiles analyzed by three-component analysis reveal down-regulation of genes involved in protein translation in human aging — SUPPLEMENTARY DATA 

# Longitudinal epigenetic and gene expression profiles analyzed by three-component analysis reveal down-regulation of genes involved in protein translation in human aging

## SUPPLEMENTARY DATA

- SUPPLEMENTARY DATA
- SUPPLEMENTARY DATA
- SUPPLEMENTARY DATA
- SUPPLEMENTARY DATA
- SUPPLEMENTARY DATA
- SUPPLEMENTARY DATA
- SUPPLEMENTARY DATA
- SUPPLEMENTARY DATA
- SUPPLEMENTARY DATA
