# Supplementary material for: Longitudinal epigenetic and gene expression profiles analyzed by three-component analysis reveal down-regulation of genes involved in protein translation in human aging
Source: Nucleic Acids Res. 2015 May 14;43(15):e100. doi: 10.1093/nar/gkv473 (PMC4551908; doi:10.1093/nar/gkv473)
Supplement: SUPPLEMENTARY DATA [file supp_gkv473_nar-03651-met-n-2014-File007_Revised.pdf]

Supplemental Experimental Procedures
Details of 3CA analysis algorithm

Jung et al.

1 Description of the algorithm

For the 3CA approach we start with either RNA-seq gene or transcript counts obtained by tophat or by processed, averaged probe intensities from Nimblegen arrays.

In order to assess the quality of information contained in the dataset we investigate three parameters associated with this information, namely the signal strength, the magnitude of the temporal change, and the magnitude of the intersample variation for each variable present in the dataset. The hypothesis to be tested is that ranking the information according to the constraints of having strong signal, large temporal change, and small intersample variation is an effective way of selecting the most relevant and reliable aging related information from the dataset in question. This way allows us to make the least amount of assumptions about the data which can be as different as RNA-seq or promoter Nimblegen arrays. This approach does not guarantee of course the optimal outcome for any data type but can be easily customized by redefining or expanding the constraints.

Given the typical tabular data of N samples over M variables

$$D_0, D_1 \in \mathbb{R}^{M \times N} \tag{1}$$

where D_0 is the data acquired at time t_0 and D_1 is the data acquired at time t_1, the pair (x_{ij}, y_{ij}) constructed from the elements of D_0 and D_1 respectively represents the jth sample of the ith variable obtained at time t_0 and t_1 respectively. The ith variable represents a single gene expression value or an averaged enrichment score over a representative promoter region of interest. In order to obtain pairs, we need N/2 sample pairs for matched individuals.

Equipped with this representation we also observe that it is natural to treat a paired j^{th} sample (x_{ij}, y_{ij}) of the ith variable as a vector in $\mathbb{R}^2$. This way, we can represent the data in a more convenient format

$$D = (d_{ij})_{M \times N}, \quad d_{ij} \in \mathbb{R}^2 \tag{2}$$

We proceed by obtaining the expected sample signal $\mu_i \in \mathbb{R}^2$ for the ith variable

$$\mu_i = \frac{1}{N} \sum_j d_{ij} \tag{3}$$

We use the mean here as a robust estimate of all sample pairs as with a low amount of samples (e.g. 20), we don't observe enough data for inferring outliers or cluster

Further, we identify the jth sample signal intensity with the Euclidean norm $\|d_{ij}\|_2$, and the signal intensity S_i for the ith variable with the norm of the expected sample signal

$$S_i = \|\mu_i\|_2 \tag{4}$$

As an adequate measure of the inter-sample variation V_i of the ith variable we choose the norm of the second central moment

$$V_i = \left\| \frac{1}{N} \sum_j (d_{ij} - \mu_i)^2 \right\|_2 \tag{5}$$

representing the variance for all sample pairs which we will refer to as inter-sample variance.

This parameter will allow us to penalize examples which are overall up or down-regulated but show a high variance in the selected samples.

The temporal change T_i of any i^{th} variable can be assessed by considering the difference between the components of μ_i, or, in other words, how far the vector μ_i deviates from the direction given by $(1, 1)$. This can be computed by taking the absolute value of the projection of μ_i onto the direction orthogonal to the $(1, 1)$, for example $(1, -1)$. Hence, we obtain

$$T_i = |\mu_i \cdot (1, -1)^T| \tag{6}$$

As such the temporal variation or change will be a single computed value, representing all sample pairs.

The triples (S_i, V_i, T_i) form an aggregate of points in the positive quadrant of $\mathbb{R}^3$ which, for simplicity, we normalize with the infinity norm and transform into log-scale obtaining

$$s_i = \log\left(\frac{S_i}{\max_i(S_i)}\right), \quad v_i = \log\left(\frac{V_i}{\max_i(V_i)}\right), \quad t_i = \log\left(\frac{T_i}{\max_i(T_i)}\right) \tag{7}$$

We also take some precaution making sure that the arguments of the *log* function are nonzero.

We are now in a position to be more specific about the hypothesis outlined above. Given the aggregate of points $q_i = (s_i, v_i, t_i)$ we are interested in selecting q_i in the order of their proximity to the point

$$q = (\max_i(s_i), \min_i(v_i), \max_i(t_i)) \tag{8}$$

Hence, what we are interested in is an objective function

$$f : \mathbb{R}^3 \to \mathbb{R} \tag{9}$$

that captures the distance between q_i and q. The square of the weighted Euclidean distance should suffice

$$f(q - q_i) = \mu_1(\max_i(s_i) - s_i)^2 + \mu_2(\max_i(t_i) - t_i)^2 + \mu_3(\min_i(v_i) - v_i)^2 \tag{10}$$

Here, $\mu_1, \mu_2, \mu_3 \in \mathbb{R}^+$ are the weights that reflect relative importance of the components. These weights can be obtained from training data if it is available, or set by a domain expert to match the desired phenomenological and experimental setup. In the absence of any relevant evidence, however, we have no reason to treat any of the components differently, dismissing the relative scales dictated by the data. Hence, setting $\mu_1 = \mu_2 = \mu_3 = 1$ represents the data driven default.

We will use $f(q - q_i)$ as the so called 3CA score which will take values between 0 which will be the most relevant and reliable aging related information from the dataset in question according to our hypothesis and a maximum numeric value in $\mathbb{R}^+$, defined by the data. The ranking of these obtained scores, representing the distance to 0, will give insights about the relative position of a gene or gene region in regards to the original constructed constraints. In our example they will represent the closest examples of genes or gene regions with minimal sample variance, maximal temporal change and maximal signal intensity.

2 3CA scoring implemented in Python

```
import numpy as np
import csv

def score(filename,sep=',',offset=2):
        """ offset is the number of leftmost columns to skip (ID's, region, etc.)"""
        D=[i for i in csv.reader(open(filename,'r'),delimiter=sep)]

        D_names=D.pop(0)
```

```
Eind=[i for i,name in enumerate(D_names[offset:]) if 'E' in name]
Lind=[i for i,name in enumerate(D_names[offset:]) if 'L' in name]
D_ID=[i[:offset] for i in D]
D=np.array([i[offset:] for i in D],dtype=float)

Ex=(np.mean(D[:,Eind],axis=1),np.mean(D[:,Lind],axis=1))
V=np.sqrt(np.std(D[:,Eind],axis=1)**2+np.std(D[:,Lind],axis=1)**2)
S=np.sqrt(Ex[0]**2+Ex[1]**2)
T=np.sqrt(.5)*abs(Ex[0]-Ex[1])

smax=lambda x: np.max(np.log(x/max(x)))-np.log(x/max(x))
smin=lambda x: np.log(x/max(x))-np.min(np.log(x/max(x)))

I=np.where((V>0)*(S>0)*(T>0))[0]
objective_f=np.sqrt(smax(S[I])**2+smax(T[I])**2+smin(V[I])**2)

return S,V,T,objective_f,I
```
