# Supplementary material for: Longitudinal epigenetic and gene expression profiles analyzed by three-component analysis reveal down-regulation of genes involved in protein translation in human aging
Source: Nucleic Acids Res. 2015 May 14;43(15):e100. doi: 10.1093/nar/gkv473 (PMC4551908; doi:10.1093/nar/gkv473)
Supplement: SUPPLEMENTARY DATA [file supp_gkv473_nar-03651-met-n-2014-File008.pdf]

Supplementary Figures

Jung et al.

Figure S1:

Alternative statistical models.

These models use a paired t-statistic against a background of inter-individual expression signal changes. A: Volcano plot for significant genes. B: Volcano plot for gene sets based on gene set enrichment analysis with a 5% FDR level cutoff C: Hierarchical clustering separates intra-individual (EL) from inter-individual (EELL) expression changes with a maximal age difference of 2 years.

Figure S2:

Single component contribution to the 3CA score.

We analyzed the top 200 3CA-ranked genes (top 200), the top 200 genes with randomly permutated samples (shuffled), and randomly chosen 200 genes (random). We calculated the inverse ranks for all component scores and then calculated the percentage for each gene (see Supplementary Experimental Procedures). The contribution of temporal changes is roughly equal for all selected categories.

Figure S3:

RT-PCR analysis of the elastin (*ELN*) gene.

ELN is downregulated in almost all sample pairs. The expression ratio of the older versus the younger sample is shown. Data was normalized to *GAPDH*.

Figure S4:

IGV browser views of the *HOXB* cluster.

A. Snapshot of normalized RNA-seq signals of the genomic region spanning the *HOXB* cluster. B. Snapshot of methylated DNA enrichment at the *HOXB* cluster and validation of DNA methylation status at the promoter region of *HOXB3* by COBRA assay. The asterisks indicate a non-specific band.

Figure S5:

IGV browser views of the *HOXD* cluster and verification by bisulfite sequencing.

A. Snapshot of methylated DNA enrichment at the *HOXD* cluster. B. Validation of DNA methylation status at the *HOXD10* gene by COBRA assay (blue box in panel A). C. Validation of DNA methylation status at the *HOXD11* gene by bisulfite sequencing (orange box in panel A).

Figure S6:

3CA-guided GSEA.

First, 3CA score distributions for the top functional terms were identified for epigenetic sets, as derived from DAVID analysis, and gene sets derived from those terms were constructed. The GSEA scores from the significant pre-ranked temporal scores of the epigenetic marks define gene sets, which were in turn tested with GSEA for all remaining data sets (e.g. “translation” in the graph description means all genes mapped to the functional terms “translation” identified with DAVID analysis for the top ranked 3CA genes with age-related DNA methylation changes in promoter regions). Though all gene sets were significant for the 3CA scores, they did not all show a clear tendency for up- or down-regulation. Top-scoring groups of functionally related genes were enriched in translation and changed DNA methylation with age (A), mitochondrion and changed H3K4me3 with age (B), DNA repair and changed H3K4me3 with age (C), cancer and changed H3K4me3 with age (D).

Figure S7:

3CA-defined variance, temporal distance and total scores for CpG islands (CGI) and surrounding shore regions.

Data shown is for H3K4 trimethylation (A, B and C), H3K27 trimethylation (D, E and F) and DNA methylation (G, H and I). Parameter values were calculated for 500 bp windows, spanning at least 4 probes.

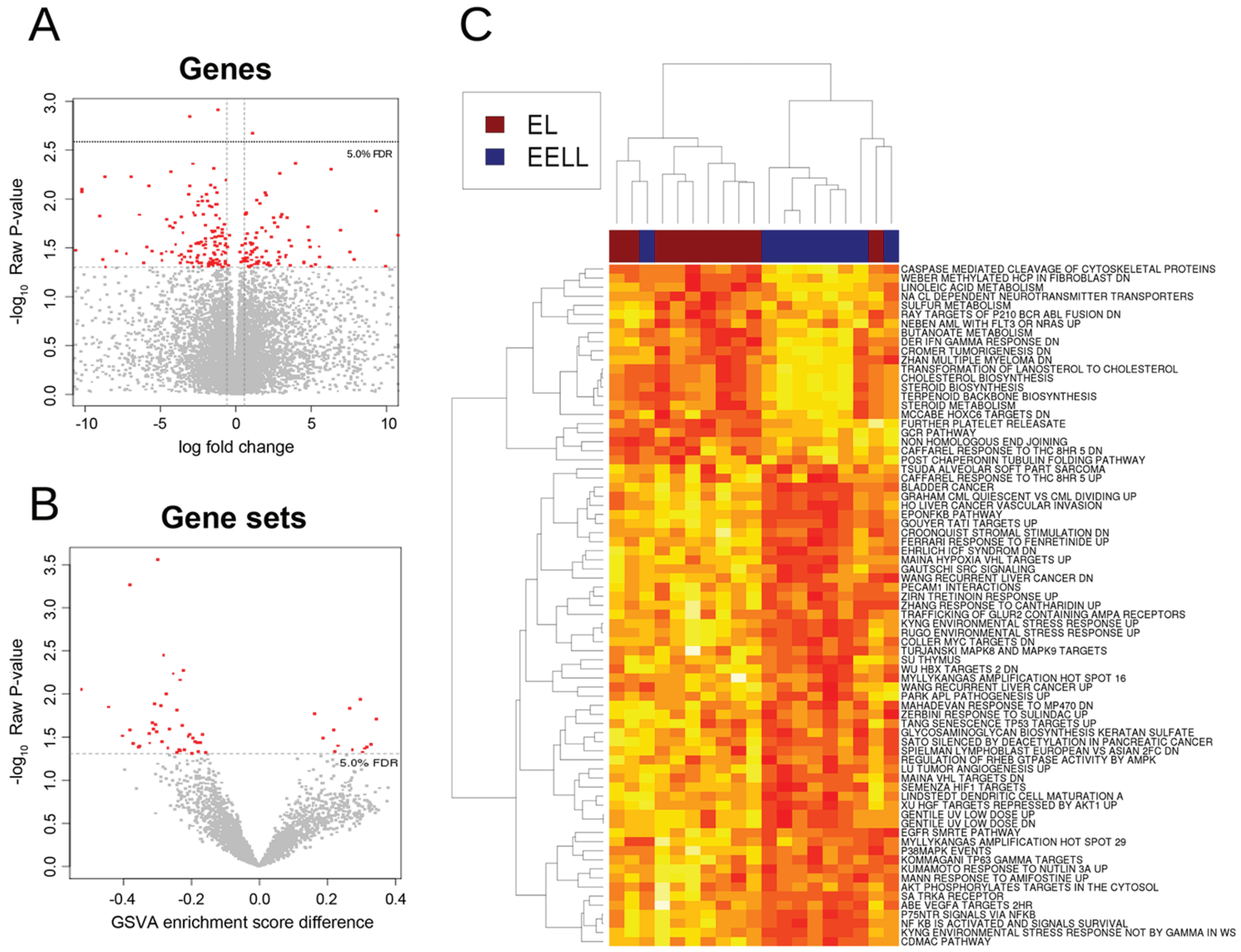
A
Genes
Raw P-value
-log10
5.0% FDR
log fold change
-10
-5
0
5
10
0.0
0.5
1.0
1.5
2.0
2.5
3.0
B
Gene sets
Raw P-value
-log10
5.0% FDR
GSVA enrichment score difference
-0.4
-0.2
0.0
0.2
0.4
0.0
0.5
1.0
1.5
2.0
2.5
3.0
3.5
C
EL
EELL
CASPASE MEDIATED CLEAVAGE OF CYTOSKELETAL PROTEINS
WEBER METHYLATED HCP IN FIBROBLAST DN
LINOLEIC ACID METABOLISM
NA CL DEPENDENT NEUROTRANSMITTER TRANSPORTERS
SULFUR METABOLISM
RAY TARGETS OF P210 BCR ABL FUSION DN
NEBEN AML WITH FLT3 OR NRAS UP
BUTANOATE METABOLISM
DER IFN GAMMA RESPONSE DN
CROMER TUMORIGENESIS DN
ZHAN MULTIPLE MYELOMA DN
TRANSFORMATION OF LANOSTEROL TO CHOLESTEROL
CHOLESTEROL BIOSYNTHESIS
STEROID BIOSYNTHESIS
TERPENOID BACKBONE BIOSYNTHESIS
STEROID METABOLISM
MCCABE HOXC6 TARGETS DN
FURTHER PLATELET RELEASATE
GCR PATHWAY
NON HOMOLOGOUS END JOINING
CAFFAREL RESPONSE TO THC 8HR 5 DN
POST CHAPERONIN TUBULIN FOLDING PATHWAY
TSUDA ALVEOLAR SOFT PART SARCOMA
CAFFAREL RESPONSE TO THC 8HR 5 UP
BLADDER CANCER
GRAHAM CML QUIESCENT VS CML DIVIDING UP
HO LIVER CANCER VASCULAR INVASION
EPONFKB PATHWAY
GOUYER TATI TARGETS UP
CROONQUIST STROMAL STIMULATION DN
FERRARI RESPONSE TO FENRETINIDE UP
EHRLICH ICF SYNDROM DN
MAINA HYPOXIA VHL TARGETS UP
GAUTSCHI SRC SIGNALING
WANG RECURRENT LIVER CANCER DN
PECAM1 INTERACTIONS
ZIRN TRETINOIN RESPONSE UP
ZHANG RESPONSE TO CANTHARIDIN UP
TRAFFICKING OF GLUR2 CONTAINING AMPA RECEPTORS
KYNG ENVIRONMENTAL STRESS RESPONSE UP
RUGO ENVIRONMENTAL STRESS RESPONSE UP
COLLER MYC TARGETS DN
TURJANSKI MAPK8 AND MAPK9 TARGETS
SU THYMUS
WU HBX TARGETS 2 DN
MYLLYKANGAS AMPLIFICATION HOT SPOT 16
WANG RECURRENT LIVER CANCER UP
PARK APL PATHOGENESIS UP
MAHADEVAN RESPONSE TO MP470 DN
ZERBINI RESPONSE TO SULINDAC UP
TANG SENESCENCE TP53 TARGETS UP
GLYCOSAMINOGLYCAN BIOSYNTHESIS KERATAN SULFATE
SATO SILENCED BY DEACETYLATION IN PANCREATIC CANCER
SPIELMAN LYMPHOBLAST EUROPEAN VS ASIAN 2FC DN
REGULATION OF RHEB GTPASE ACTIVITY BY AMPK
LU TUMOR ANGIOGENESIS UP
MAINA VHL TARGETS DN
SEMENZA HIF1 TARGETS
LINDSTEDT DENDRITIC CELL MATURATION A
XU HGF TARGETS REPRESSED BY AKT1 UP
GENTILE UV LOW DOSE UP
GENTILE UV LOW DOSE DN
EGFR SMRTE PATHWAY
MYLLYKANGAS AMPLIFICATION HOT SPOT 29
P38MAPK EVENTS
KOMMAGANI TP63 GAMMA TARGETS
KUMAMOTO RESPONSE TO NUTLIN 3A UP
MANN RESPONSE TO AMIFOSTINE UP
AKT PHOSPHORYLATES TARGETS IN THE CYTOSOL
SA TRKA RECEPTOR
ABE VEGFA TARGETS 2HR
P75NTR SIGNALS VIA NFKB
NF KB IS ACTIVATED AND SIGNALS SURVIVAL
KYNG ENVIRONMENTAL STRESS RESPONSE NOT BY GAMMA IN WS
CDMAC PATHWAY

Fig. S1

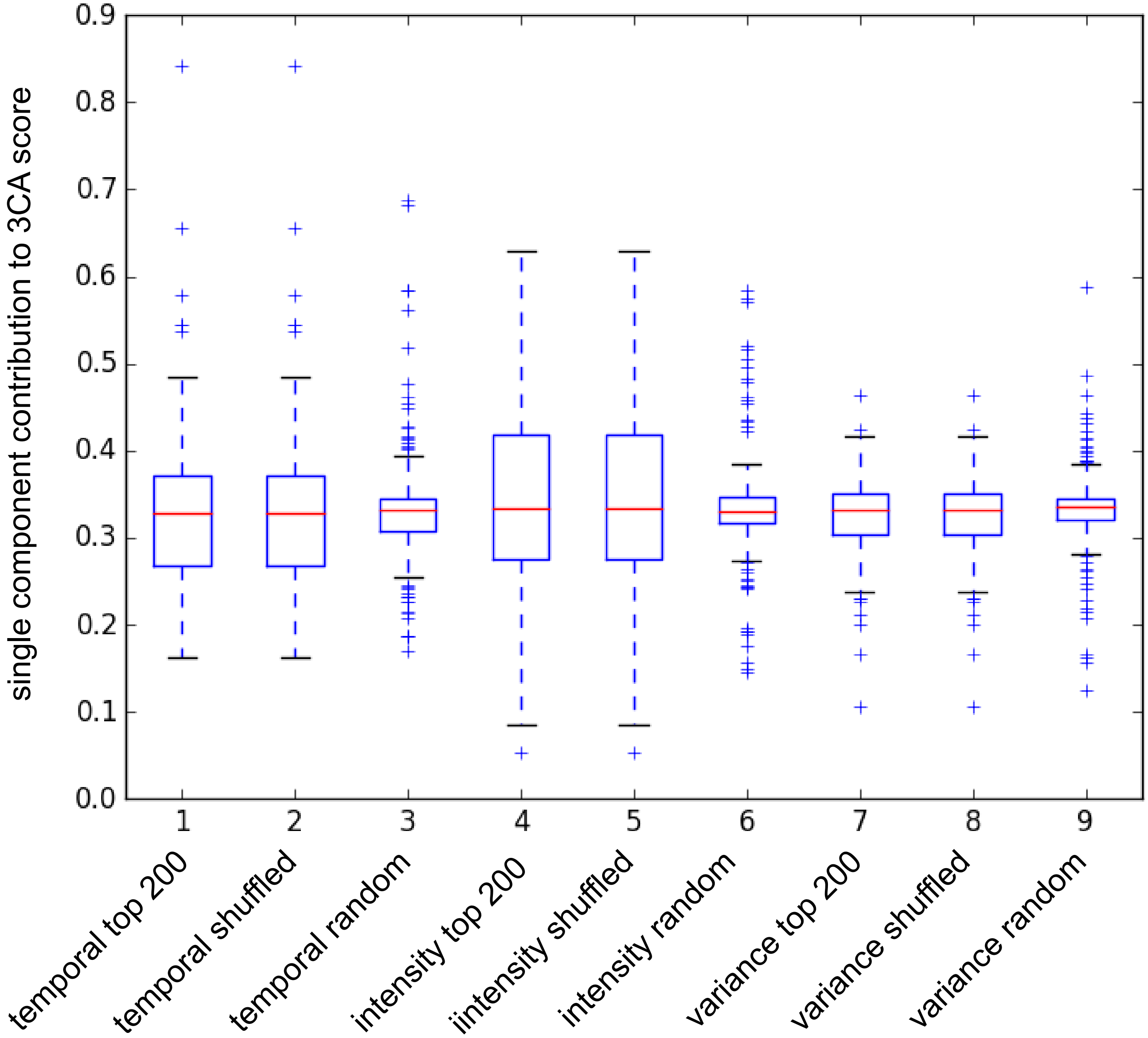
single component contribution to 3CA score
0.0
0.1
0.2
0.3
0.4
0.5
0.6
0.7
0.8
0.9
1
2
3
4
5
6
7
8
9
temporal top 200
temporal shuffled
temporal random
intensity top 200
iintensity shuffled
intensity random
variance top 200
variance shuffled
variance random

Fig. S2

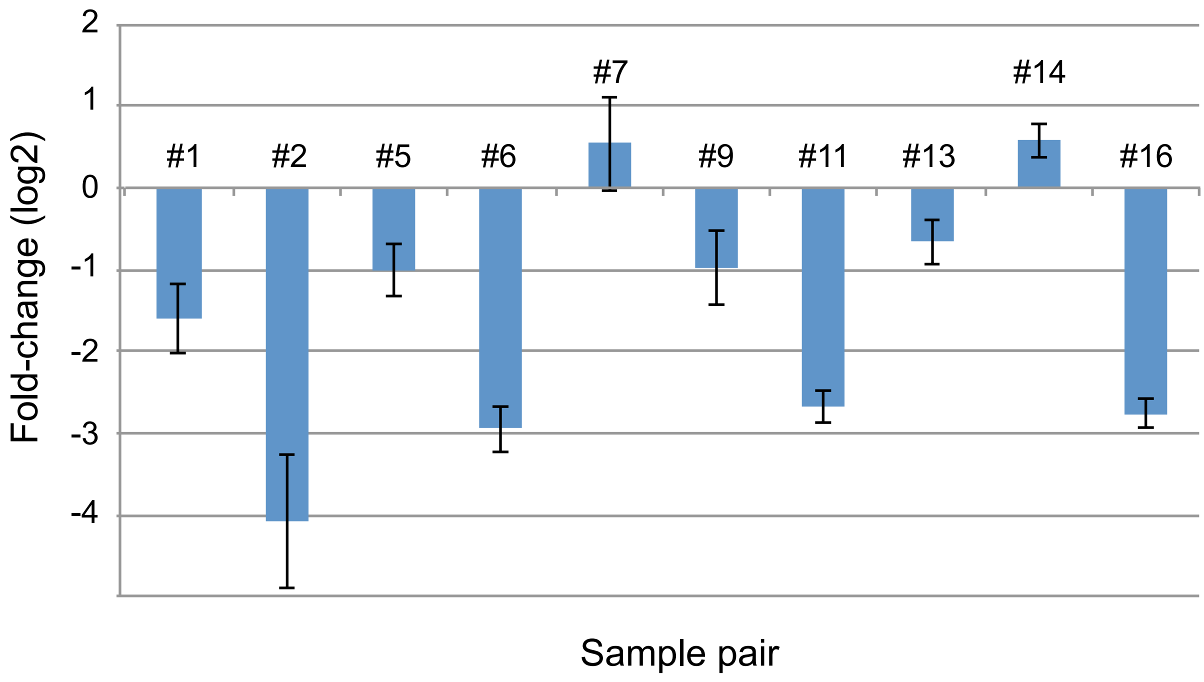

Fig. S3

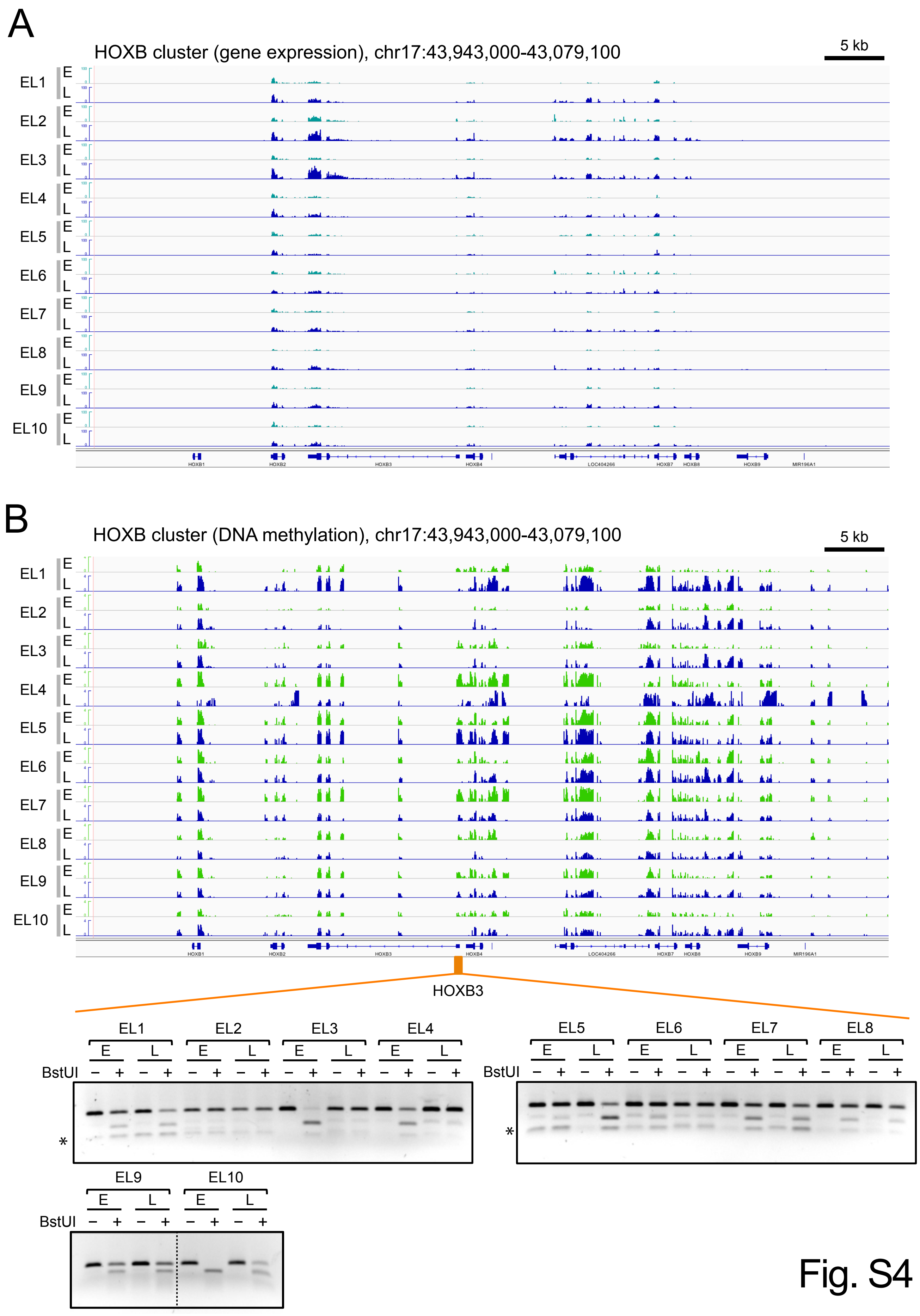
A
HOXB cluster (gene expression), chr17:43,943,000-43,079,100
5 kb
EL1
EL2
EL3
EL4
EL5
EL6
EL7
EL8
EL9
EL10
E
L
B
HOXB cluster (DNA methylation), chr17:43,943,000-43,079,100
5 kb
HOXB3
BstUI
EL1
EL2
EL3
EL4
EL5
EL6
EL7
EL8
EL9
EL10
*

Fig. S4

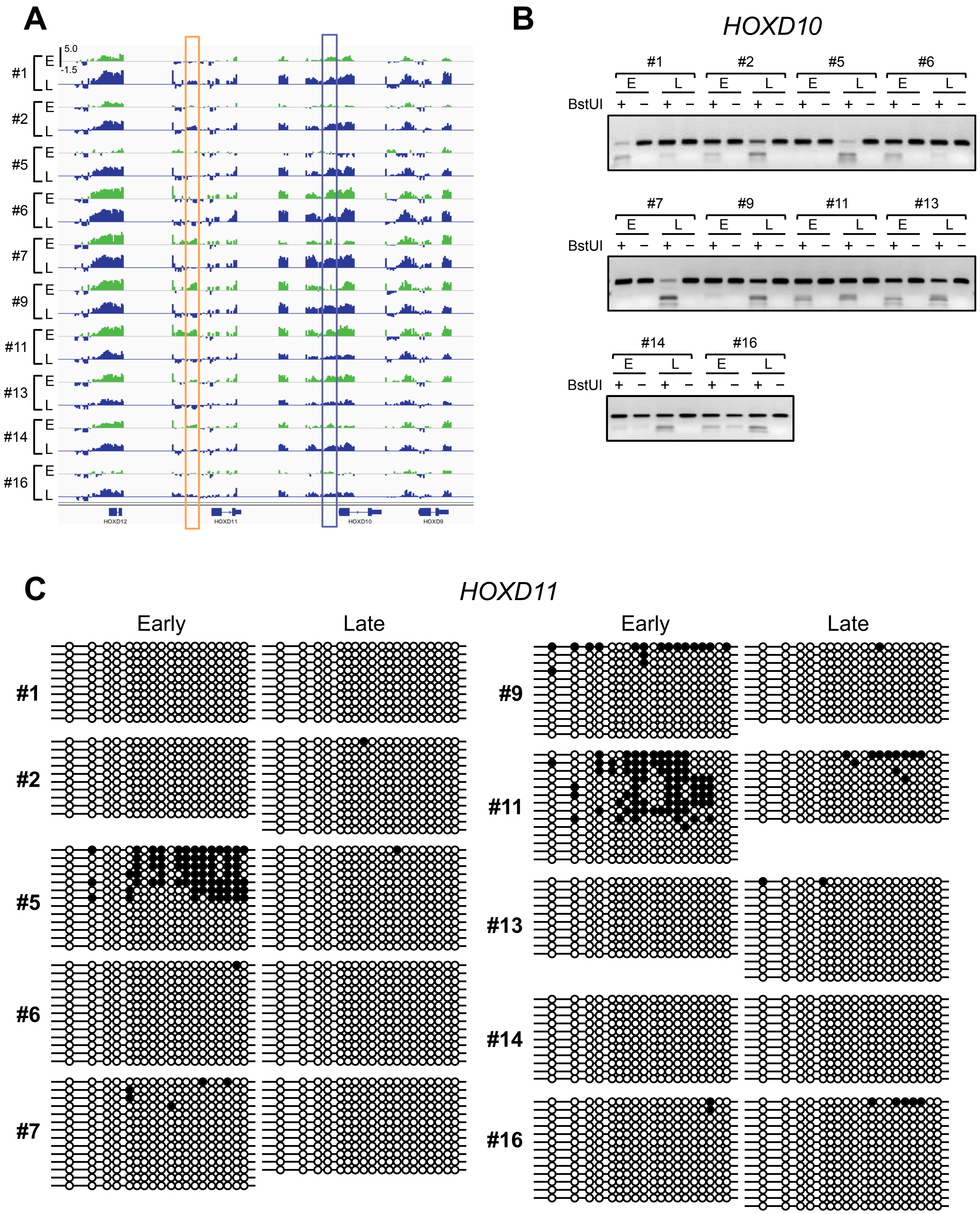
A
5.0
-1.5
#1
#2
#5
#6
#7
#9
#11
#13
#14
#16
E
L
HOXD12
HOXD11
HOXD10
HOXD9
B
HOXD10
#1
#2
#5
#6
#7
#9
#11
#13
#14
#16
E
L
BstUI
+
–
C
HOXD11
Early
Late
#1
#2
#5
#6
#7
#9
#11
#13
#14
#16

Fig. S5

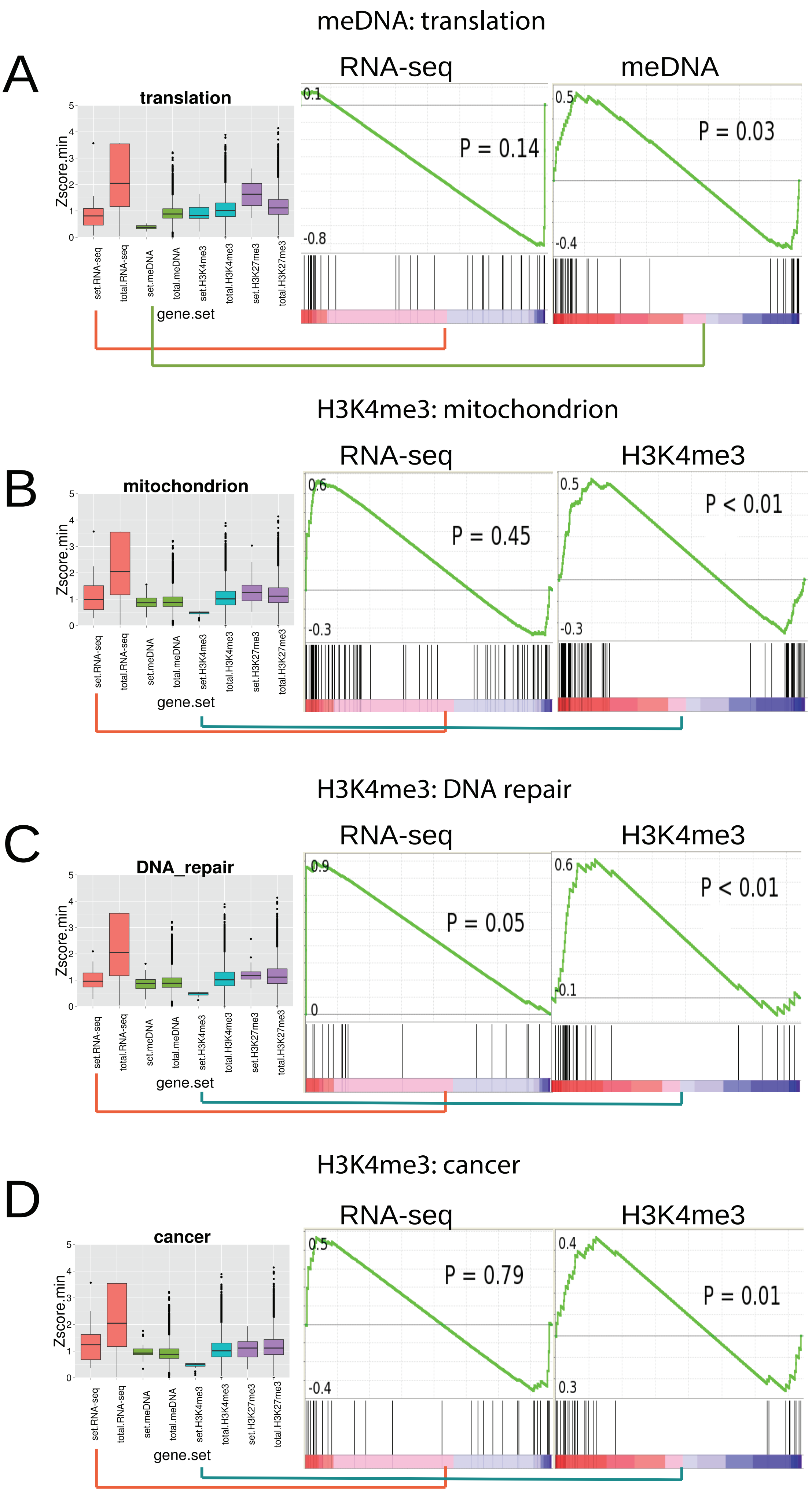

meDNA: translation
A
translation
RNA-seq
meDNA
P = 0.14
P = 0.03
Zscore.min
gene.set
set.RNA-seq
total.RNA-seq
set.meDNA
total.meDNA
set.H3K4me3
total.H3K4me3
set.H3K27me3
total.H3K27me3
H3K4me3: mitochondrion
B
mitochondrion
RNA-seq
H3K4me3
P = 0.45
P < 0.01
H3K4me3: DNA repair
C
DNA_repair
RNA-seq
H3K4me3
P = 0.05
P < 0.01
H3K4me3: cancer
D
cancer
RNA-seq
H3K4me3
P = 0.79
P = 0.01

Fig. S6

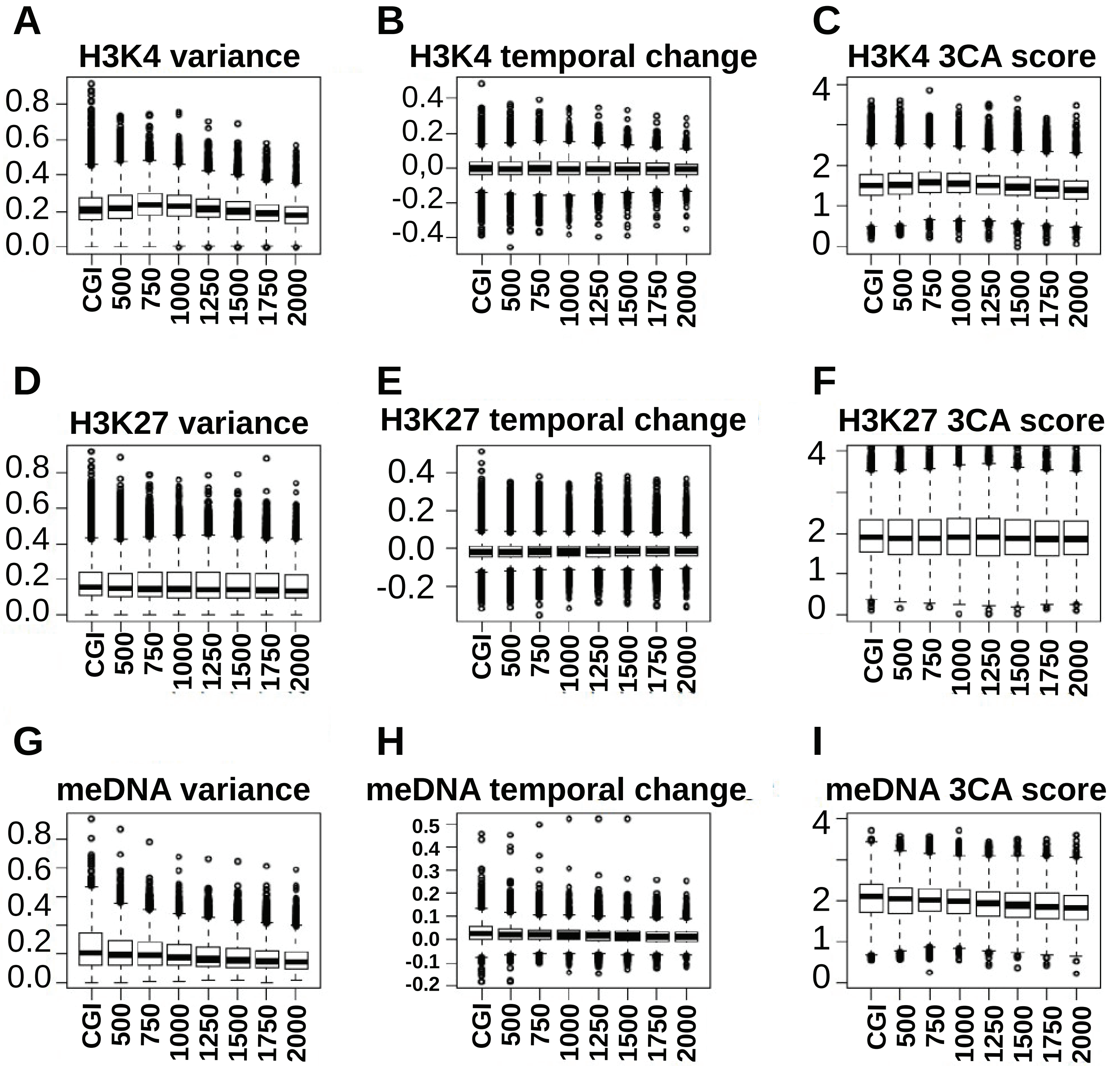
A
H3K4 variance
B
H3K4 temporal change
C
H3K4 3CA score
D
H3K27 variance
E
H3K27 temporal change
F
H3K27 3CA score
G
meDNA variance
H
meDNA temporal change
I
meDNA 3CA score
CGI
500
750
1000
1250
1500
1750
2000

Fig. S7
